# Efficient strategy for the molecular diagnosis of intractable early-onset epilepsy using targeted gene sequencing

**DOI:** 10.1186/s12920-018-0320-7

**Published:** 2018-02-01

**Authors:** John Hoon Rim, Se Hee Kim, In Sik Hwang, Soon Sung Kwon, Jieun Kim, Hyun Woo Kim, Min Jung Cho, Ara Ko, Song Ee Youn, Jihun Kim, Young Mock Lee, Hee Jung Chung, Joon Soo Lee, Heung Dong Kim, Jong Rak Choi, Seung-Tae Lee, Hoon-Chul Kang

**Affiliations:** 1 Department of Laboratory Medicine, Severance Hospital, Yonsei University College of Medicine, 50-1 Yonsei-ro, Seodaemun-gu, Seoul, 03722 South Korea; 2 Divison of Pediatric Neurology, Department of Pediatrics, Severance Children’s Hospital, Yonsei University College of Medicine, Epilepsy Research Institute, 50-1 Yonsei-ro, Seodaemun-Gu, Seoul, 03722 South Korea; 3 Brain Korea 21 PLUS Project for Medical Science, Yonsei University College of Medicine, Seoul, South Korea; 4 Department of Pediatrics, Gangnam Severance Hospital, Yonsei University College of Medicine, Seoul, South Korea; 5 Department of Pediatrics, National Health Insurance Corporation Ilsan Hospital, Goyang, South Korea

**Keywords:** Diagnostic yield, Early-onset epilepsy, Next-generation sequencing

## Abstract

**Background:**

We intended to evaluate diagnostic utility of a targeted gene sequencing by using next generation sequencing (NGS) panel in patients with intractable early-onset epilepsy (EOE) and find the efficient analytical step for increasing the diagnosis rate.

**Methods:**

We assessed 74 patients with EOE whose seizures started before 3 years of age using a customized NGS panel that included 172 genes. Single nucleotide variants (SNVs) and exonic and chromosomal copy number variations (CNVs) were intensively examined with our customized pipeline and crosschecked with commercial or pre-built software. Variants were filtered and prioritized by in-depth clinical review, and finally classified according to the American College of Medical Genetics and Genomics guidelines. Each case was further discussed in a monthly consensus meeting that included the participation of all laboratory personnel, bioinformaticians, geneticists, and clinicians.

**Results:**

The NGS panel identified 28 patients (37.8%) with genetic abnormalities; 25 patients had pathogenic or likely pathogenic SNVs in 17 genes including *SXTBP1* (*n* = 3), *CDKL5* (*n* = 2), *KCNQ2* (*n* = 2), *SCN1A* (*n* = 2), *SYNGAP1* (*n* = 2), *GNAO1* (*n* = 2), *KCNT1* (*n* = 2), *BRAT1*, *WWOX*, *ZEB2*, *CHD2*, *PRICKLE2*, *COL4A1*, *DNM1*, *SCN8A*, *MECP2*, *SLC9A6* (*n* = 1). The other 3 patients had pathogenic CNVs (2 duplications and 1 deletion) with varying sizes (from 2.5 Mb to 12 Mb). The overall diagnostic yield was 37.8% after following our step-by-step approach for clinical consensus.

**Conclusions:**

NGS is a useful diagnostic tool with great utility for patients with EOE. Diagnostic yields can be maximized with a standardized and team-based approach.

**Electronic supplementary material:**

The online version of this article (10.1186/s12920-018-0320-7) contains supplementary material, which is available to authorized users.

## Background

Early-onset epilepsy (EOE) in infancy or early childhood is often a devastating form of epilepsy that is associated with severe cognitive impairment. Etiologies remain unidentified despite comprehensive structural, metabolic, immunologic, infection, and genetic investigations due to their heterogeneity [1]. Recently, several studies demonstrated the usefulness of targeted next-generation sequencing (NGS) for diagnosing EOE using a genetic approach [2–6]. Previous studies have reported increased diagnostic yields with NGS panels compared to those obtained with other genetic tests [2, 7]. NGS strategies have revolutionized epilepsy genomic research by increasing the detection of de novo variants using targeted sequencing and whole-exome sequencing (WES).

Nevertheless, clinical implications of diagnostic NGS panel for EOE are currently limited. Previous studies focused on identifying the efficacy of NGS in large, multicenter patient cohorts. Limited clinical descriptions of patients were provided. Clinical questions regarding the identification of patients who are most likely to show positive genetic results, and strategies to increase the diagnostic yields of NGS panel still remain to be explored.

Here, we used a customized NGS panel to clinically and genetically investigate a highly selected group of patients who developed EOE in infancy or early childhood (≤3 years of age). All analytical steps were performed within our center according to a systematized diagnostic pipeline. Our goal was to optimize the clinical use of NGS panel for EOE by finding the most important analytical step that determines the diagnosis rate and identifying patients who are most likely to show genetic abnormalities.

## Methods

### Patients and clinical information

A total of 74 unrelated pediatric patients with EOE without a known cause were recruited from the epilepsy clinic in Severance Children’s Hospital from March 2015 to May 2016. As a nationwide referral center for EOE, patient population generally includes severe epilepsy patients with unknown causes. All patients met the following criteria: (1) seizure onset before the age of 3 years; (2) multiple epileptiform discharges with severely disorganized background activity on electroencephalography (EEG); (3) diagnosed with drug-resistant epilepsy and progressive developmental delay or with a known epileptic encephalopathy syndrome; (4) no structural lesion detected with brain magnetic resonance imaging (MRI); (5) no metabolic abnormalities; (6) no abnormalities detected with previous genetic tests; and (7) offspring of asymptomatic Korean parents. For the diagnosis of specific epilepsy syndrome, patients were classified according to the 2010 International League Against Epilepsy classification [8] and previous diagnostic criteria.

All EEGs were reviewed by one of four pediatric epileptologists (S.H.K, H.K, J.S.L, and H.D.K). All EEG recordings were obtained according to the 10–20 international system, using a 21-channel digital EEG system (Xltek, Natus Medical Incorporated, San Carlos, CA, USA). Data were recorded using a sampling rate of 200 Hz with filter settings of 1–70 Hz. Brain MRI was performed according to standard epilepsy protocols, including oblique coronal view on T2-weighted and fluid-attenuated inversion recovery at 1.5−3.0 Tesla. Investigations for metabolic disorders included plasma amino acid analysis, urine organic acid analysis, homocysteine level, acylcarnitine profile (total and free carnitine levels), blood gas analysis, and serum lactate, pyruvate, and ammonia. Previous genetic tests in each patient varied but usually included chromosomal analysis, Sanger sequencing for specific genes such as *MECP2*, and multiplex ligation-dependent probe amplification (MLPA) using the SALSA MLPA P245 Microdeletion Syndrome kit (MRC Holland, Amsterdam, The Netherlands), which were mainly performed before referral from various other hospital to Severance Children’s Hospital.

For clinical analysis, previous seizure history was reviewed in detail for the age of seizure onset, seizure types, history of neonatal seizures, history of status epilepticus, and febrile seizures. Information regarding admission to the neonatal intensive care unit (NICU), developmental state, and premature deaths were collected. This study was approved by the Institutional Review Board of Severance Hospital (IRB 4–2016-0080). Informed consent was obtained from patients or their parents.

### Targeted gene sequencing using NGS panel

For the customized NGS panel, we selected 172 candidate genes implicated in EOE based on an extensive literature review and the Online Mendelian Inheritance in Man (OMIM) database (http://www.ncbi.nlm.nih.gov/omim) (listed in online Additional file 1: Table S1). These genes include genes related to not only EOE, but also epilepsies of inborn errors of metabolism and conditions that are indications or contraindications of the ketogenic diet.

Genomic DNAs were extracted from leukocytes of whole blood samples using the QIAamp Blood DNA mini kit (Qiagen, Hilden, Germany) according to the manufacturer’s instructions. Intact DNA was quantified and adjusted to a concentration of 5 ng/μL using a Qubit 2.0 Fluorometer (Invitrogen, Waltham, MA, USA) and the Qubit dsDNA HS assay kit (Invitrogen). Precapture libraries were constructed according to the manufacturer’s sample preparation protocol. Each patient’s genomic DNA was fragmented to a median size of 300 base pair (bp). The DNA fragments were end-repaired, phosphorylated, and adenylated on the 3′ ends. The index adaptors were ligated to the repaired ends, DNA fragments were amplified, and fragments of 200−500 bp were isolated. Pooled libraries were sequenced using a MiSeq sequencer (Illumina, San Diego, CA, USA) and the MiSeq Reagent Kit v2 (300 cycles).

### NGS data analysis

Data analysis was performed primarily through our custom pipeline. Briefly, raw sequence data were mapped to GRCh37 (hg19) using the Burrows-Wheeler Aligner algorithm, followed by removal of duplicate reads, realignment of insertions and deletions, base quality recalibration, and variant calling using the Genome Analysis Toolkit (GATK). Small nucleotide variants called by GATK were further crosschecked with BaseStudio software (Illumina). Every variant that was suspected to be pathogenic, likely pathogenic, or variant of unknown significance (VUS) was confirmed by visual inspection of the bam file using Integrated Genomics Viewer version 2.3 (IGV; Broad Institute, Cambridge, MA, USA). Quality metrics were calculated for each sample using the FastQC software and TEQC package.

Split-read-based detection of large insertions and deletions was conducted using the Pindel and Manta algorithms, and both results were finally crosschecked. Read-depth-based detection of structural rearrangements was conducted using the ExomeDepth software. Chromosomal copy number variations (CNVs) detected by ExomeDepth were further crosschecked using our custom pipeline; this retrieved base-level depth-of-coverage for each bam file using SAMtools software and normalized the depths against those of other samples in the same batch. We performed off-target analysis of chromosomal copy number alterations using the CopywriteR software.

The following databases were used for variant annotation: OMIM, Human Gene Mutation Database (HGMD), ClinVar, dbSNP, 1000 Genome, Exome Aggregation Consortium (ExAC), Exome Sequencing Project, and Korean Reference Genome Database (KRGDB). The pathogenicity of missense variants was predicted using five in silico prediction algorithms, including Sorting Tolerant from Intolerant (SIFT), Polymorphism Phenotyping v2 (PolyPhen-2), MutationTaster, MutationAssessor, and Functional Analysis through Hidden Markov Models (FATHMM) implemented in dbNSFP version 3.0a. Effects on splicing were predicted using the SPIDEX version 1.0 and dbscSNV version 1.1.

### Annotation and interpretation of variants

**Fig. 1:**
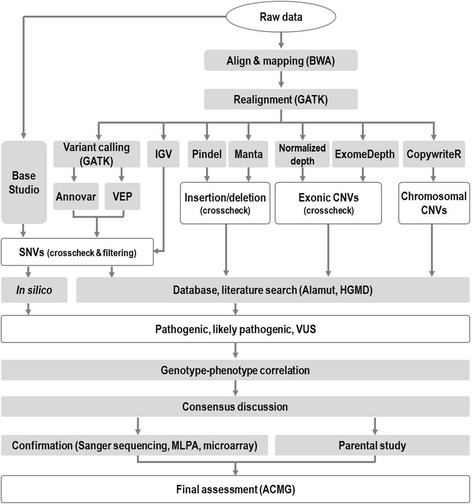
Flow chart of our bioinformatics pipeline and variant classification results with interpretation

Identified variants were described according to nomenclature recommendations of the Human Genome Variation Society (http://www.hgvs.org/mutnomen). The interpretation of variants followed the 5-tier classification system recommended by the American College of Medical Genetics and Genomics and the Association for Molecular Pathology (ACMG/AMP) [9] using a step-by-step approach (Fig. 1). Briefly, ACMG guideline defined 28 criteria that address evidence such as population data, case-control analyses, functional data, computational predictions, allelic data, segregation studies, and de novo observations. We evaluated all the possible components in every patient by three-step analyses. First, variants were filtered by their frequencies in population control databases, including ExAC (non-TCGA dataset; frequencies were calculated according to ethnic subgroups), ESP6500, 1000 Genomes Project, and KRGDB. To maximize the diagnostic yield, variants with a minor allele frequency (MAF) greater than 5% in any of the population subgroups rather than conventional 1% criteria were classified as absolutely benign, whereas those that were absent from the general population were considered to have moderate evidence as pathogenic. Second, literature and database searches for previous reports and functional studies were performed using the Alamut Visual 2.6 software (Interactive Biosoftware, France) and HGMD professional database. When all in silico analyses showed consistent predictions, the results were regarded as evidence of benign or pathogenic variants.

### Phenotype review and consensus discussion

The last step involved genetic specialists or laboratory physicians presenting a preliminary report to the patient’s attending physicians or pediatric epileptologists, which listed all possible pathogenic variants, likely pathogenic variants, and VUSs. The clinicians performed an in-depth review of each patient’s phenotype and gave an opinion from their point of view. Each case was further reviewed and discussed in a monthly consensus meeting that included the participation of all laboratory personnel, bioinformaticians, geneticists, and clinicians. When pathogenic or likely pathogenic variants were consistent with the patient’s phenotype, final validation using other confirmatory assays and a parental study was planned. VUSs, especially missense variants, were prioritized according to population frequency, ACMG score, and the patient’s clinical phenotype. A parental study was scheduled to detect de novo occurrence for the candidate pathogenic, likely pathogenic, and VUS variants in all available trios.

### Confirmation using other methods

For small nucleotide variations, pathogenic and likely pathogenic variants as well as VUSs needing parental study were examined using Sanger sequencing on a 3730 DNA Analyzer with the BigDye Terminator v3.1 Cycle Sequencing kit (Applied Biosystems, Foster City, CA, USA). Sequencing data were aligned against appropriate reference sequences and analyzed using the Sequencher 5.3 software (Gene Codes Corp., Ann Arbor, MI, USA).

Large exonic deletions and duplications were confirmed using the MLPA kit (MRC Holland). Chromosomal copy number alterations were confirmed using the Infinium CytoSNP 850 K array (Illumina) and BlueFuse Multi software (Illumina).

### Statistical analysis

To compare clinical presentations among subgroups according to epilepsy syndrome type or presence of definitive genetic causes revealed by this study, Fisher’s exact test, Chi-square test, and independent *t*-test were utilized for comparisons of numerical and categorical variables. Bonferroni correction was utilized for multiple testing correction. Statistical analyses were computed using the PASW statistics software (version 18.0, SPSS Inc., Chicago, IL, USA). *P*-value <0.05 was considered statistically significant.

## Results

### Demographics and clinical characteristics of patients

Demographics and clinical characteristics of all patients are summarized in online Additional file 1: Table S2. Mean age of seizure onset was 7.5 ± 7.8 months (standard deviation). Seizures started before the age of 1 year in 85.1% of patients (63/74). Global developmental delay was observed in 83.8% of patients (62/74). Among them, 17 (23.0%) patients could not make eye contact. Epileptic spasms (70.3%) were the most common seizure type, followed by generalized (33.8%) and focal (21.6%) seizure types. Two patients had all three seizure types. Many patients had a remarkable perinatal history, which included admission to the NICU in 23.0% of patients (17/74) and a history of neonatal seizures in 15.0% of patients (11/74). Six (8.1%) of the 74 patients had a history of status epilepticus, and two (2.7%) had unexpected premature deaths. The causes of death were pneumonia and sudden infantile death syndrome. The epilepsy syndrome was diagnosed most commonly as infantile spasm (IS) (*n* = 51), followed by Dravet syndrome (*n* = 2), malignant migrating focal seizures in infancy (MMFI) (*n* = 1), and Doose syndrome (*n* = 1).

### NGS run and quality metrics

Quality metrics of NGS runs for all patients are summarized in online Additional file 1: Table S3. On average, more than 8.7 million reads were sequenced per sample, and approximately 8.0 million reads (93.5%) were mapped on references. The average horizontal coverage, which was interpreted as percentage of regions with more than 20× coverage, was 99.8%.

### Spectrum and statistics of pathogenic variants

**Fig. 2:**
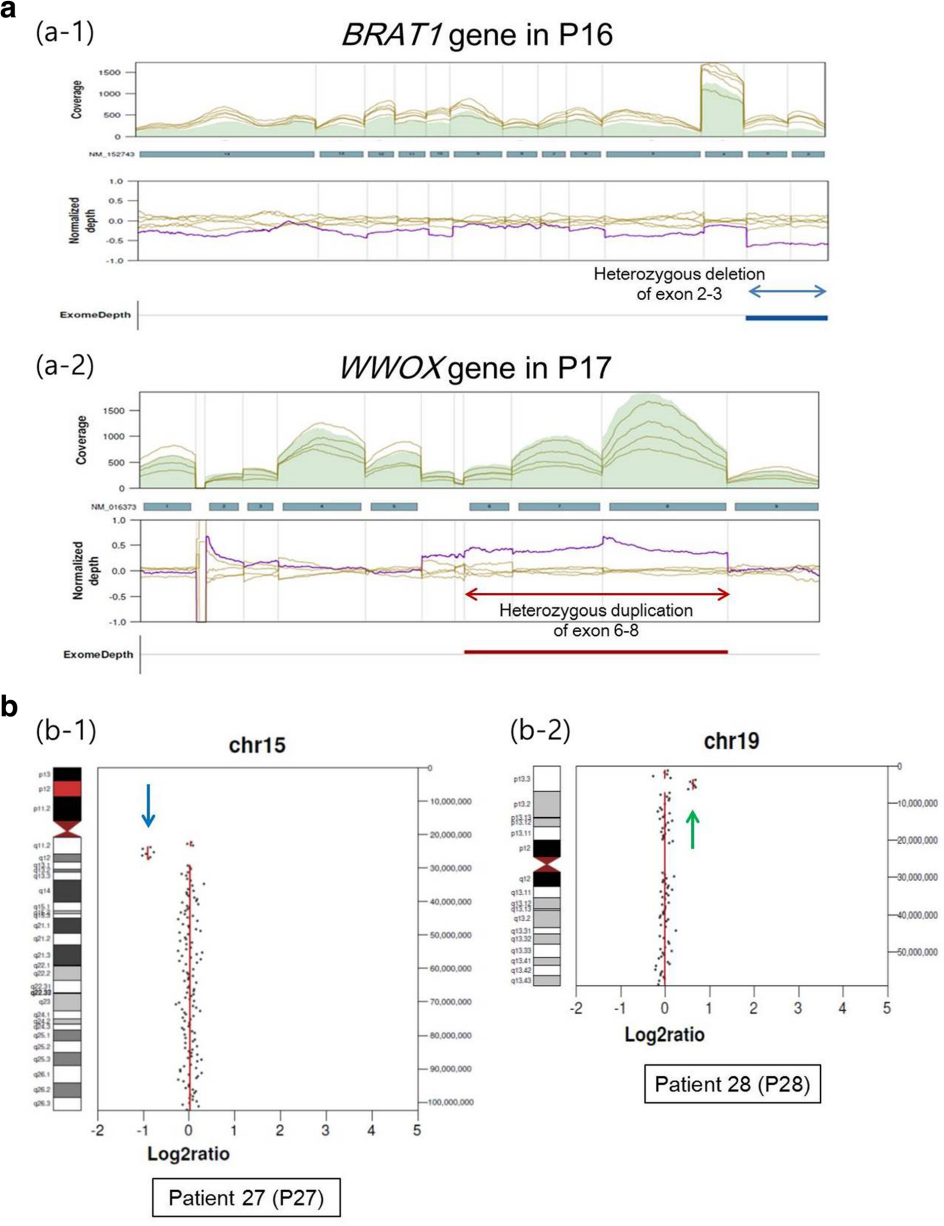
Profiles of mutations detected in our study. **a** Exonic deletions and duplications detected by relative-depth comparisons. **a-1** Heterozygous deletion involving exons 2−3 in the *BRAT1* gene of patient 16 (P16), (**a-2**) heterozygous duplication involving exons 6−8 in the *WWOX* gene in patient 17 (P17). **b** Examples of chromosomal copy number alterations detected by off-target analysis of targeted next-generation sequencing results. **b-1** Heterozygous deletion of the 15q11.2 region (downward arrow) observed in patient 27. **b-2** Heterozygous duplication of the 19p13.3 region (upward arrow) observed in patient 28

Among the 28 patients with pathogenic or likely pathogenic variants, 25 had single nucleotide variants (SNVs) or nucleotide/exon deletions in 17 epilepsy-associated genes (Table 1), and 3 had deletion or duplication of large genomic segments (Table 2). Among the SNVs, missense variants (*n* = 14) were the most common type, followed by nonsense (*n* = 5), frameshift (*n* = 4), and splice-site (*n* = 2) variants (Table 1). Two patients (P16 and P17) were compound heterozygous for one SNV and one exon-level deletion/duplication (Table 1, Fig. 2a). The most commonly affected gene was *STXBP1*.

**Table 1:** Results of mutation analysis in 25 patients with pathogenic or likely pathogenic mutations involving single nucleotide or several exons according to ACMG guideline

Patient	Gene	Inheritance	NM	Nucleotide change	Amino acid change	Zygosity	ACMG classification	ACMG evidence components	Origin of variant
P1	*STXBP1*	AD	NM_003165.3	c.733C > G	p.His245Asp	Heterozygosity	Pathogenic	PS2, PM2, PM5, PP3, PP4	de novo
P2	*STXBP1*	AD	NM_003165.3	c.874C > T	p.Arg292Cys	Heterozygosity	Pathogenic	PS1, PS2, PM2, PP4	de novo
P3	*STXBP1*	AD	NM_003165.3	c.1216C > T	p.Arg406Cys	Heterozygosity	Pathogenic	PS1, PS2, PM2, PM5, PP3, PP4	de novo
P4	*CDKL5*	XD	NM_003159.2	c.511 T > A	p.Tyr171Asn	Heterozygosity	Pathogenic	PS2, PM2, PM5, PP3, PP4	de novo
P5	*CDKL5*	XD	NM_003159.2	c.282 + 1G > A	splice site	Heterozygosity	Pathogenic	PVS1, PS2, PM2, PP4, PP5	de novo
P6	*KCNQ2*	AD	NM_172107.2	c.917C > T	p.Ala306Val	Heterozygosity	Pathogenic	PS2, PM2, PP2, PP3, PP4, PP5	de novo
P7	*KCNQ2*	AD	NM_172107.2	c.593G > A	p.Arg198Gln	Heterozygosity	Pathogenic	PS2, PM2, PP2, PP3, PP4, PP5	de novo
P8	*SCN1A*	AD	NM_001165963.1	c.5068_5069delinsG	p.Ser1690AlafsTer25	Heterozygosity	Pathogenic	PVS1, PM2, PP4	NA
P9	*SCN1A*	AD	NM_001165963.1	c.1209dupT	p.Val404CysfsTer46	Heterozygosity	Pathogenic	PVS1, PM2, PP4	NA
P10	*SYNGAP1*	AD	NM_006772.2	c.980 T > C	p.Leu327Pro	Heterozygosity	Likely pathogenic	PS2, PM2, PP3, PP4, PP5	de novo
P11	*SYNGAP1*	AD	NM_006772.2	c.1735C > T	p.Arg579Ter	Heterozygosity	Pathogenic	PVS1, PM2, PP4, PP5	NA
P12	*GNAO1*	AD	NM_020988.2	c.118G > T	p.Gly40Trp	Heterozygosity	Pathogenic	PS2, PM2, PM5, PP3, PP4	de novo
P13	*GNAO1*	AD	NM_020988.2	c.155A > C	p.Gln52Pro	Heterozygosity	Likely pathogenic	PS2, PM2, PP3, PP4	de novo
P14	*KCNT1*	AD	NM_020822.2	c.2800G > A	p.Ala934Thr	Heterozygosity	Likely pathogenic	PS1, PM2, PP4	NA
P15	*KCNT1*	AD	NM_020822.2	c.1038C > G	p.Phe346Leu	Heterozygosity	Likely pathogenic	PS2, PM2, PP2, PP4	de novo
P16	*BRAT1*	AR	NM_152743.3	c.1576C > T	p.Gln526Ter	Heterozygosity	Likely pathogenic	PVS1, PM2, PP4	maternal inheritance
P16	*BRAT1*	AR	NM_152743.3	exon 2–3 deletion	_	Heterozygosity	Likely pathogenic	PM2, PM3, PP4, PP5	NA
P17	*WWOX*	AR	NM_016373.2	c.1060C > T	p.Gln354Ter	Heterozygosity	Pathogenic	PVS1, PM2, PM3, PP4	maternal inheritance
P17	*WWOX*	AR	NM_016373.2	exon 6–8 duplication	_	Heterozygosity	Likely pathogenic	PM2, PM3, PP4, PP5	paternal inheritance
P18	*ZEB2*	AD	NM_014795.3	c.1956C > A	p.Tyr652Ter	Heterozygosity	Pathogenic	PVS1, PS1, PM2, PP4	NA
P19	*CHD2*	AD	NM_001271.3	c.1269dupA	p.Glu424ArgfsTer3	Heterozygosity	Pathogenic	PVS1, PM2, PP4	NA
P20	*PRICKLE2*	AD	NM_198859.3	c.2129_2147del	p.Arg710LeufsTer2	Heterozygosity	Likely pathogenic	PVS1, PM2	NA
P21	*COL4A1*	AD	NM_001845.4	c.2897G > A	p.Gly966Glu	Heterozygosity	Likely pathogenic	PS2, PM2, PP2, PP3, PP4	de novo
P22	*DNM1*	AD	NM_004408.2	c.1195A > G	p.Arg399Gly	Heterozygosity	Likely pathogenic	PS2, PM2, PP2, PP3, PP4	de novo
P23	*SCN8A*	AD	NM_014191.3	c.782G > T	p.Cys261Phe	Heterozygosity	Likely pathogenic	PS2, PM2, PP2, PP3, PP4	de novo
P24	*MECP2*	XD	NM_004992.3	c.1164_1207del	p.Pro389Ter	Heterozygosity	Pathogenic	PVS1, PM1, PM2, PP4	NA
P25	*SLC9A6*	XD	NM_006359.2	c.316_325 + 28del	splice site	Heterozygosity	Pathogenic	PVS1, PS2, PM2, PP4	de novo

*AD* autosomal dominant, *XD* X dominant, *AR* autosomal recessive, *VUS* variant of unknown significance, *PVS* pathogenic very strong, *PS* pathogenic strong, *PM* pathogenic moderate, *PP* pathogenic supporting, *NA* not available

**Table 2:** Results of mutation analysis in 3 patients with pathogenic or likely pathogenic copy number variations

Patient	Affected region	Duplication/Deletion	Size	Major genes involved in the region	Additional study	Result of the additional study	Zygosity	Suspected syndrome	Phenotypic correlation
P26	14q11.2-q12	Duplication	12 Mb	*FOXG1, CHD8, SUPT16H*	array CGH	arr 14q11.2(20,528,528–32,297,926)×3	Heterozygosity	Not available^a^	Early onset of infantile spasms,Developmental delay
P27	15q11.2	Deletion	3.5 Mb	*UBE3A, GABRB3, SNRPN*	MLPA	Deletion of maternal allele	Heterozygosity	Angelman syndrome	Distinctive electro-encephalography pattern^c^
P28	19p13.3	Duplication	2.5 Mb	Not specific epilepsy-associated genes	array CGH	arr 19p13.3(3,462,574–6,583,781)×3	Heterozygosity	19p13.3 microduplication syndrome^b^	Distinctive facial dysmorphism

^a^ No definitive syndrome was suggested until the two most recent literatures [22, 23]

^b^ New microduplication syndrome was suggested by Carmen et al. [24]

^c^ High amplitude rhythmic 4–6 Hz activity, prominent in the occipital or frontal regions with spikes

For CNVs, abnormalities were found in heterozygote form for three different chromosomes in three patients (Table 2, Fig. 2b). Two patients had seizure type of epileptic spasms, whereas the seizure type of the other patient was unknown. The affected segment sizes varied from 2.5 to 12 Mb, and all CNVs were confirmed using methylation-specific MLPA or array comparative genomic hybridization. In patient P27, methylation-specific MLPA confirmed deletion of maternal alleles in the 15q11.2 region, which contains *SNRPN*, *UBE3A*, and *GABRB3*. The phenotype and distinctive EEG pattern of patient P27 were compatible with Angelman syndrome, which is prolonged runs of high amplitude rhythmic 2–3 Hz activity predominantly over the frontal regions with superimposed interictal epileptiform discharges and high amplitude rhythmic 4–6 Hz activity prominent in the occipital regions with spikes.

Overall, 16/51 (31.4%) patients with IS and 8/19 (42.1%) patients with unknown EOE could be genetically diagnosed with our epilepsy NGS panel (online Additional file 1: Table S4). The most common type of mutation in IS was missense in *STXBP1*. Both patients with Dravet syndrome harbored frameshift mutations in *SCN1A* and one patient with Doose syndrome had a *CHD2* mutation.

### Clinical factors associated with genetic abnormalities

When all patients with EOE were assessed together, we did not find statistically significant differences in gender, age of seizure onset, and history of neonatal seizures between the patients with sufficient genetic cause group identified by NGS and the patients with negative results group, except for history of febrile seizures (28.6% vs. 6.5%) (online Additional file 1: Table S5). However, when only patients who had IS were assessed, severe clinical outcomes were more prevalent in patients with genetic cause than in patients without identified genetic cause. Patients with an identified genetic cause were more likely to never develop eye contact (50.0% vs. 17.1%, *P* = 0.02) (Table 3). Also more patients had an earlier seizure onset than in patients without a detected pathogenic mutation, suggesting more severe epilepsy in this group of patients with genetic causes (3.2 ± 3.0 months vs. 6.2 ± 4.4 months, *P* = 0.02).

**Table 3:** Clinical and demographic information of the patients with infantile spasm

	Total number	Group 1 (Patients with positive genetic cause, % within group 1)	Group 2 (Patients without positive genetic cause, % within group 2)	*P*-value
Number of subjects	51	16	35	
Female	23	10 (62.5)	13 (37.1)	0.09
Seizure onset (months)				1.00
0 < *n* ≤ 12 months	49	16 (100)	33 (94.3)	
12 months < *n* ≤ 24 months	2	0	2 (5.7)	
24 months < *n* ≤ 36 months	0	0	0	
mean age (± standard deviation)		3.2 (± 3.0)	6.2 (± 4.4)	0.02
Global developmental delay	41	14 (87.5)	27 (77.1)	0.47
Absence of eye contact	14	8 (50.0)	6 (17.1)	0.02
Neonatal seizures	9	5 (31.3)	4 (11.4)	0.12
Febrile seizures	2	2 (12.5)	0	0.09
Status epilepticus	1	0	1 (2.9)	1.00
Premature death	2	1 (6.3)	1 (2.9)	0.53
NICU stay	20	7 (43.8)	8 (22.9)	0.19

## Discussion

Using a customized NGS panel, more than one-third of unknown EOEs could be diagnosed genetically. Given the high diagnostic yield, NGS panel could be a powerful diagnostic tool for clinical practices with EOE patients. We developed a diagnostic process that pediatric epileptologists and bioinformaticians can easily implement in a clinical setting. Furthermore, we provide a simplified flowchart that other epilepsy centers can follow by showing benefits acquired at each step sequentially. All analytical steps were carefully controlled, and diagnostic rates at all stages were reviewed.

In this study, more than 170 genes were examined to include as many genes as possible in order not to underestimate the genetic contribution to epilepsy. As anticipated, a few well-known genes accounted for the majority of observed abnormalities. Single-gene mutations accounted for 89.2% of all abnormalities detected; these included 16 mutations (57.1%) in major affected genes in our study (*STXBP1*, *CDKL5*, *KCNQ2*, *SCN1A*, *KCNT1*, *SYNGAP1*, *MECP2*, and *GNAO1*) and 9 mutations (32.1%) in various genes (*BRAT1, WWOX, ZEB2, CHD2, PRICKLE2, COL4A1, DNM1, SCN8A*, and *SLC9A6*). Although *PRICKLE2* is controversial to be defined as epilepsy-associated gene based on previous studies, our case might serve as an additional evidence for pathogenicity. Overall, 90.2% (156) of the 172 genes in our panel showed no abnormalities. These results suggest that the currently recognized causative genes of EOE can only account for a very small fraction of the total causes of EOE, and more genes can cause EOE than have been previously reported.

Our results are consistent with those of previous studies [2, 7]. A recent Chinese study used an NGS panel for EOE that included 17 genes, and successfully diagnosed 32% (56/175) of patients [10]. A recent Japanese study examined 35 genes and diagnosed 23% (12/53) of patients [11]. Except for a few commonly mutated genes, most pathogenic variants in other genes with minor frequency seem to be rare causes of EOE. The diagnostic yield of NGS panel appeared to be determined by the number of commonly mutated genes included in the panel rather than the total number of genes in the panel [12]. This result might indicate that specific genes which have been repeatedly reported by multiple centers in multiple patients who share similar clinical phenotypes including epilepsy deserve more cautious and detailed analysis than other genes. Clinical use of NGS panel for EOE will be maximized when the incidence of all causative genes is determined and considered, rather than blindly increasing the number of genes in a panel with a hope to proportionally increase the diagnostic rate.

Patient inclusion criteria play a critical role in determining the diagnostic rate of NGS panel. The diagnostic rate increases when the study includes only narrowly classified patients with severe clinical phenotypes. In our EOE study, patients with febrile seizures and poor eye contact were more likely to exhibit genetic abnormalities. This phenomenon was more clearly demonstrated in the IS patient subgroup, and patients were more likely to exhibit genetic abnormalities if they had early onset epileptic spasms or poor eye contact (*P* < 0.05). These results suggest that patients with unexplained IS are more likely to exhibit abnormalities in the NGS panel if they experience seizures at younger ages and have more phenotypic traits, such as absence of eye contact.

Although intractable epilepsy is a condition that can occur at any age, it occurs most frequently in infancy and early childhood. In this study, we included only patients who met the following criteria: (1) seizures before 3 years of age and (2) progressive refractory EOE of unknown origin or well-known refractory epilepsy syndrome. We believe that our study was performed with the strict inclusion criteria, including age limits. In previous studies, the diagnostic rate of NGS panel tended to negatively correlate with age limits. The diagnostic rates increased to 32% when only patients under 6 months of age were included [10], and declined to 12% when all patients under 18 years of age were included [13]. However, it is possible that selection bias of patients with severe phenotypes might have caused relatively high diagnotic yield in this study.

Many studies have proved the clinical usefulness of NGS panel, and more epilepsy centers will use NGS panel for the management of EOE. An objective and standardized interpretation of data is critical for academic communication and validation [14]. Here, we show the importance of analyzing the genomic data systematically using a standardized approach. We found that accurate interpretation of gene abnormalities requires the following three crucial components: (1) population data and database review; (2) computational data, allelic data, and literature review; and (3) detailed clinical review, family study, and consensus discussions. Our analysis process presented the diagnostic yield of the NGS panel to be 37.8%. It should also be emphasized that consensus discussions can occur only when bioinformaticians, geneticists, and clinical neurologists closely communicate. We conducted multi-disciplinary team meetings every month, supported by frequent online and offline communications. Our study emphasizes the importance of collaborative work for correct interpretation of NGS results. The benefits of team interpretations of genomic data in rare diseases have been previously emphasized [15]. In this study, we demonstrate that team interpretation can be particularly beneficial for managing patients with EOE whose known genes and genetic inheritance patterns are diverse.

In our study, exonic and chromosomal CNVs were identified in 2.7% (2/74) and 4.1% (3/74) of patients, respectively. The importance of CNVs in epilepsy has been confirmed in previous studies [16–18]. With the rapid evolution of NGS technologies and algorithms, CNV detection using NGS could replace traditional methods. Exonic CNVs can be detected using relative depth comparisons, and whole chromosomal CNVs can be detected using off-target analysis. All identified CNVs were confirmed by MLPA or chromosomal microarrays, as well as phenotype comparison with patients of similar affected regions such as distinctive facial dysmorphism (P28 in Table 2). We also concluded that off-target analysis could be an effective and practical way to detect chromosomal CNVs, and targeted NGS testing may serve as a candidate first-line genomic test to replace chromosomal microarrays.

Unfortunately, we failed to identify causal mutations for approximately two-thirds of patients. Unknown genes may be causative factors in these patients [19, 20]. The fact that several NGS or WES studies have reported similar percentages of negative results [5, 10, 21] indicate that other genetic mechanisms including epigenetics and somatic mutation may play a role.

## Conclusions

In conclusion, this study demonstrated that the targeted NGS panel has excellent diagnostic performance and great utility for managing patients with EOE. Accurate and appropriate interpretation with extensive CNV detection strategies increased the diagnostic yield of targeted NGS.

## Additional file

Additional file 1: Table S1. List of 172 targeted genes included in the epilepsy panel. **Table S2.** Clinical and demographic information of the patients. **Table S3.** Quality control matrices of NGS test results for all patients in this study. **Table S4.** Diagnostic yield of targeted NGS according to types of epilepsy syndrome.** Table S5.** Clinical factors associated with genetic abnormalities. (XLSX 31 kb)

## Abbreviations

ACMG: American College of Medical Genetics and Genomics
AMP: Association for Molecular Pathology
bp: Base pair
CNV: Copy number variation
EEG: Electroencephalography
EOE: Early-onset epilepsy
ExAC: Exome Aggregation Consortium
FATHMM: Functional Analysis through Hidden Markov Models
GATK: Genome analysis toolkit
HGMD: Human Gene Mutation Database
MAF: Minor allele frequency
MLPA: Multiplex ligation-dependent probe amplification
MMFI: Malignant migrating focal seizures in infancy
MRI: Magnetic resonance imaging
NGS: Next generation sequencing
NICU: Neonatal intensive care unit
OMIM: Online Mendelian Inheritance in Man
PolyPhen-2: Polymorphism Phenotyping v2
SIFT: Sorting Tolerant from Intolerant
SNV: Small nucleotide variant
VUS: Variant of unknown significance
WES: Whole-exome sequencing
